# Unravelling cognitive frailty: perceptions, misconceptions, and the path to prevention

**DOI:** 10.1093/geront/gnag112

**Published:** 2026-05-26

**Authors:** Gerda Prakopimaite, Magdalena Pfaff, Eddy J Davelaar, Lisa Quadt, Malgorzata Raczek, Naji Tabet, Alan J Gow, Carol Holland, Dorina Cadar

**Affiliations:** CEDAR Lab, Centre for Dementia Studies, Department of Clinical Neuroscience, Brighton and Sussex Medical School, University of Sussex, Sussex, United Kingdom; Division of Psychology and Language Science, University College London, London, United Kingdom; CEDAR Lab, Centre for Dementia Studies, Department of Clinical Neuroscience, Brighton and Sussex Medical School, University of Sussex, Sussex, United Kingdom; Centre for Cognition, Computation and Modelling, Department of Psychological Sciences, Birkbeck, University of London, London, United Kingdom; CEDAR Lab, Centre for Dementia Studies, Department of Clinical Neuroscience, Brighton and Sussex Medical School, University of Sussex, Sussex, United Kingdom; CEDAR Lab, Centre for Dementia Studies, Department of Clinical Neuroscience, Brighton and Sussex Medical School, University of Sussex, Sussex, United Kingdom; CEDAR Lab, Centre for Dementia Studies, Department of Clinical Neuroscience, Brighton and Sussex Medical School, University of Sussex, Sussex, United Kingdom; The Ageing Lab, Department of Psychology and Centre for Applied Behavioural Sciences, Heriot-Watt University, Edinburgh, United Kingdom; Centre for Ageing Research, Faculty of Health and Medicine, Lancaster University, Lancashire, United Kingdom; CEDAR Lab, Centre for Dementia Studies, Department of Clinical Neuroscience, Brighton and Sussex Medical School, University of Sussex, Sussex, United Kingdom

**Keywords:** Frailty, Dementia, Age in place

## Abstract

**Background and Objectives:**

As populations age, extending healthy life expectancy and reducing morbidity are priorities. Cognitive frailty, the co-occurrence of cognitive impairment and physical frailty without dementia, may offer a window for prevention. However, there is limited evidence on how cognitive frailty is understood, which may undermine recognition and risk reduction. This study provides new insights into how cognitive frailty is conceptualized across public and professional contexts by exploring perceptions, causes, and risk factors of cognitive frailty to inform public health and clinical practice.

**Research Design and Methods:**

We conducted 22 semi-structured interviews with members of the public and healthcare professionals. Interviews examined perceived definitions, symptoms, and risk factors. Data were analyzed using reflexive thematic analysis.

**Results:**

Three analytic domains were identified: conceptualizations, manifestations, and risk factors. Participants often conflated cognitive frailty with cognitive impairment or described it as an early stage of dementia. The physical frailty aspect was under-recognized, particularly among public participants. When presented with the international definition, many reframed cognitive frailty as a transitional and potentially preventable state. Healthcare professionals articulated broader symptom profiles and more integrated risk frameworks, whereas the public emphasized observable physical change. Across both groups, mood changes, social withdrawal, and unhealthy lifestyle were viewed as central risks, though their interplay was described with varying detail.

**Discussion and Implications:**

Inconsistent understanding of cognitive frailty may hinder early recognition and prevention. Clearer operational definitions, supportive policy, and targeted education are needed to strengthen detection, risk reduction, and intervention strategies that protect independence in later life.

## Introduction

With increasing life expectancy, age-related conditions such as dementia and frailty are becoming more prevalent, posing substantial personal, societal, and economic challenges. At a global level, population aging and extended longevity have intensified focus on functional health and independence in later life (United Nations, 2024; World Health Organization, 2024). In relation to dementia, given its progressive and historically incurable nature, interventions have focused on symptom management (Emmady & Tadi, 2022; Profyri et al., 2022), although more recently, novel disease-modifying treatments have been introduced (Belder et al., 2023). To enable intervention at a stage when treatment is still likely to be effective, early identification of individuals at risk of dementia and related functional decline is crucial not only for pharmacological approaches but also for facilitating timely, targeted non-pharmacological interventions, including those addressing both cognitive and physical vulnerability. Within this context, increasing attention has focused on identifying intermediate states of vulnerability that capture the interplay between cognitive and physical decline.

Within this broader effort to identify early risk states, cognitive frailty has emerged as a key marker of vulnerability (Kelaiditi et al., 2013). Internationally, cognitive frailty is defined as the co-occurrence of physical frailty and cognitive impairment in the absence of dementia (Kelaiditi et al., 2013). Its prevalence varies widely, ranging from approximately 1% in younger-old community-dwelling adults aged 65–70 years to over 20% in older clinical or institutional populations, based on international population-based and clinical studies of older adults, conducted in countries including France, Japan, Canada, Italy, and Singapore (Panza et al., 2018; Ruan et al., 2015). Such variation reflects differences in age, setting, and diagnostic criteria, but consistently points to cognitive frailty as a significant public health concern. Importantly, cognitive frailty is best understood not as a prodromal dementia diagnosis, but as a heterogeneous risk state associated with multiple possible trajectories. Although it is associated with increased risk of dementia, evidence from longitudinal and meta-analytic studies indicates that individuals with cognitive frailty may follow diverse trajectories, including progression to disability, loss of independence, institutionalization, or mortality, rather than dementia alone (Bu et al., 2021; Zhang et al., 2022). Framing cognitive frailty solely through a dementia-centered lens risks obscuring its broader functional consequences and may limit opportunities for earlier, multidomain intervention focused on maintaining independence and quality of life.

To further situate this construct, although cognitive frailty may overlap with mild cognitive impairment (MCI), the two constructs are conceptually distinct. MCI primarily reflects cognitive decline without major functional impairment, and meta-analytic evidence from predominantly high-income-country cohorts suggests that progression rates to dementia vary widely across populations, settings, and diagnostic criteria (Mitchell & Shiri-Feshki, 2009). In contrast, cognitive frailty integrates both cognitive vulnerability and physical frailty, reflecting a broader and potentially more modifiable risk profile. While physical frailty and cognitive impairment independently increase dementia risk (Rivan et al., 2024), cognitive frailty represents a synergistic vulnerability in which deficits across both domains exacerbate one another (Zheng et al., 2020). This interaction accelerates functional decline and is linked with increased risks of disability, institutionalization, and mortality beyond either condition alone (Bu et al., 2021; Shimada et al., 2018; Zhang et al., 2022). Taken together, these distinctions highlight cognitive frailty as a multidomain construct positioned at the intersection of physical frailty and cognitive impairment, rather than a simple extension of either.

Evidence suggests that early identification and multidomain intervention may help delay progression toward dementia, disability, and loss of independence, although effects vary by cognitive frailty subtype (Kelaiditi et al., 2013; Ruan et al., 2015; Zheng et al., 2022). Interventions targeting physical activity, nutrition, cognitive stimulation, psychosocial well-being, social connectedness, and supportive environments have shown promise (Aguilar-Navarro et al., 2025; Fowler-Davis et al., 2024a, 2024b; Hodgson et al., 2023; Holland et al., 2024; Murukesu et al., 2020; Ponvel et al., 2021; Sugimoto et al., 2022). However, their effectiveness depends partly on whether cognitive frailty is recognized, understood, and acted upon by healthcare professionals and the public.

Challenges remain in screening and diagnosing cognitive frailty in clinical practice. Although Kelaiditi et al. (2013) proposed combining cognitive screening with comprehensive physical assessment, existing tools are often underused due to time constraints, lack of standardization, and concerns about diagnostic precision (Nader et al., 2023; Sugimoto et al., 2018). Cognitive screening instruments such as the Mini-Mental State Examination are widely considered inadequate for detecting MCI, further complicating early identification (Breton et al., 2019; Mitchell, 2009). While recent efforts have focused on developing more precise diagnostic protocols and self-assessment tools for cognitive frailty (Kelaiditi et al., 2013; Nader et al., 2023; Rivan et al., 2024; Tseng et al., 2019), even improved tools are likely to have limited impact if the condition itself is not framed consistently or communicated clearly to patients, families, and professionals.

The evolving understanding of dementia throughout the twentieth century (Ballenger, 2006a, 2006b, 2017) illustrates the close relationship between societal perceptions, diagnostic practices, and access to intervention (Ballenger, 2017). As Rosenberg famously argued, “In some ways, the disease does not exist until we have agreed that it does, by perceiving, naming, and responding to it” (Rosenberg et al., 1992). In a similar way, how cognitive frailty is named and understood may shape whether early signs are recognized, whether risk is interpreted as modifiable, and whether preventive opportunities are pursued.

Public and professional perceptions of cognitive frailty are likely to influence whether it is recognized, discussed, and acted upon. Frailty is often viewed as an inevitable part of aging and associated with negative connotations, which may reduce engagement in health-promoting behaviors and discourage help-seeking (Parish et al., 2019; Schoenborn et al., 2018; Shafiq et al., 2023). Similarly, diagnoses of MCI and frailty have been associated with poorer quality of life and depressive symptoms (Guzmán et al., 2021; Sánchez-García et al., 2017). Yet cognitive frailty remains largely confined to aging-related research, limiting its visibility in broader healthcare and public contexts. Healthcare professionals may also avoid the term frailty because of its potentially discouraging implications (Griffin et al., 2024). These issues highlight the need to examine how cognitive frailty is conceptualized in everyday and professional discourse. Therefore, this study explored how healthcare professionals and members of the general public conceptualize cognitive frailty, including how it is defined, recognized, and linked to perceived risk factors, to inform earlier recognition and more targeted prevention and intervention strategies.

## Method

### Study design

We used a qualitative design to explore perceptions of cognitive frailty among members of the general public and healthcare professionals. Semi-structured interviews were selected to capture nuanced interpretations of a concept increasingly discussed in aging research but variably understood in public and clinical discourse.

The interview guide was developed by a multidisciplinary research team with expertise in aging, cognitive frailty, and dementia. It was piloted with two individuals with lived experience of cognitive impairment or dementia to assess clarity and relevance. Following feedback, we made minor revisions, including simplifying clinical terminology, rewording selected questions, and adding prompts to support reflection across both public and professional audiences. Topic development was also informed by a biopsychosocial framework of aging, drawing on Holland et al. (2024), which emphasizes psychological, lifestyle, and socio-environmental influences on cognitive frailty.

The study was approved by the Brighton and Sussex Medical School, University of Sussex Research Ethics Committee (reference number ER/MP667/1).

### Participants and recruitment

Participants were recruited using purposive, convenience, and snowball sampling. Healthcare professionals were purposively sampled across roles relevant to frailty, cognitive impairment, and older adult care. Members of the general public were recruited to capture perspectives from adults outside the healthcare sector. Recruitment took place via Twitter/X advertisements and word of mouth. Interested individuals contacted the research team by email and were then sent a participant information sheet.

Twenty-two participants were interviewed: 12 healthcare professionals and 10 members of the general public. Inclusion criteria for both groups were age 18 years or older, English fluency, and the capacity to provide informed consent. Group-specific eligibility criteria were applied. Healthcare professionals were required to be currently working in the UK healthcare sector (including clinical roles and healthcare-facing research or service delivery roles). Members of the general public were required to have no current or prior professional experience in the healthcare sector. Participants received reimbursement for their time. Participant characteristics are presented in Table 1.

**Table 1:** Characteristics of participants.

Group	Participant number	Age	Gender	Highest level of education	Familiarity with the term “cognitive frailty”	Works with frail people
**Public**	1	23	Man	Bachelor’s degree	No	No
	2	30	Man	Master’s degree	No	No
	3	22	Man	Apprenticeship	No	No
	4	23	Man	Bachelor’s degree	No	No
	5	25	Woman	A level	No	No
	6	22	Woman	A level	No	No
	7	23	Woman	A level	No	No
	8	26	Woman	Bachelor’s degree	Yes	No
	9	30	Man	Master’s degree	Yes	No
	10	23	Man	Master’s degree	No	No
				**Occupation**		
**Healthcare professionals**	11	53	Woman	Research Coordinator	Yes	Yes
	12	42	Woman	Project Manager for dementia educational program	Yes	No
	13	37	Woman	Research fellow	Yes	Yes
	14	28	Woman	PhD student and Clinician in Old Age Psychiatry	Yes	Yes
	15	32	Woman	Old Age Psychiatrist	Yes	Yes
	16	38	Woman	Admiral Nurse	Yes	Yes
	17	29	Woman	Clinical Research Coordinator in dementia	No	Yes
	18	53	Woman	Academic and Clinician Geriatrician	Yes	No
	19	47	Woman	Clinical Research Coordinator	Yes	Yes
	20	28	Woman	Clinical Research Coordinator	Yes	Yes
	21	33	Man	Clinical Research Fellow, medical doctor	Yes	No
	22	55	Woman	Consultant Psychiatrist	Yes	Yes

### Procedures

Interested individuals received a participant information sheet and contacted the research team by email. Eligibility screening was completed prior to scheduling an interview. Written and verbal informed consent were obtained before the interview commenced.

Interviews were conducted in person or online, lasted 30–60 min, and were audio-recorded with consent. Participants first described their understanding of cognitive frailty before receiving the international definition as a shared reference point. Interviews explored perceived definitions, manifestations, sequencing of cognitive and physical decline, and risk factors, including mood, social engagement, and lifestyle behaviors. The topic guide is provided as Supplementary Material.

### Data collection

Interviews were conducted by two researchers (M.P. and G.P.) with postgraduate training and experience in qualitative interviewing and analysis. Audio recordings were automatically transcribed, then manually checked and corrected to ensure accuracy.

Sample adequacy was guided by the principle of information power (Malterud et al., 2016), considering the focused study aim, the specificity of the participant groups, and the depth of the interview material. In keeping with reflexive thematic analysis, we did not pursue “saturation” as a procedural endpoint. Rather, iterative review indicated that later interviews largely elaborated existing patterns rather than introducing substantively new concepts relevant to the analytic domains.

### Thematic analysis

We conducted reflexive thematic analysis following Braun and Clarke (2019). This approach was selected to examine how participants construct meaning around cognitive frailty, recognizing that analysis is shaped by the interaction between participants’ accounts and the researcher’s interpretation, rather than assuming a single objective account.

Analysis used a hybrid deductive-inductive strategy. Deductively, analysis was organized around three analytic domains aligned with the study aims and interview structure: (1) conceptualizations and definitions of cognitive frailty, (2) perceived manifestations and symptom recognition, and (3) perceived risk factors. These domains served as organizing frameworks rather than themes. Within each domain, themes were developed inductively through iterative coding and reflexive engagement with the data. Theme development prioritized conceptual coherence and interpretive depth rather than frequency of occurrence.

NVivo 14 supported data management. G.P. undertook initial coding, with ongoing reflexive discussion and theme refinement with D.C. and M.P. Interviews and analyses were conducted by researchers with postgraduate training in qualitative methods and disciplinary backgrounds in cognitive aging, dementia research, and health psychology. This professional positioning informed familiarity with cognitive frailty and related clinical concepts, while reflexive discussions examined how these perspectives might shape interpretation. Analytic meetings focused on questioning assumptions, exploring alternative readings of the data, and ensuring that interpretations remained grounded in participants’ accounts rather than driven by preexisting theoretical or clinical frameworks. Consistent with reflexive thematic analysis, inter-rater reliability was not calculated, and researcher interpretation was treated as an analytic resource. Reporting followed a qualitative reporting checklist (Kmet et al., 2004).

## Results

The sample included 22 participants: 12 healthcare professionals and 10 members of the general public. Participant characteristics are presented in Table 1. Healthcare professionals ranged in age from 28 to 55 years (*M* = 39.6, *SD* = 9.8) and included 11 women and one man. Most held postgraduate degrees, and nine reported direct experience working with frail older adults. Participants from the general public were aged 22 to 30 years (*M* = 24.7, *SD* = 2.9), with six men and four women, and educational backgrounds ranging from secondary education to a master’s degree.

Reflexive thematic analysis was conducted within three analytic domains aligned with the study aims: (1) conceptualizations of cognitive frailty, (2) perceived manifestations and symptom recognition, and (3) perceived risk factors. Domains reflected the interview structure and served as organizing frameworks rather than themes. Themes were developed inductively within each domain and are illustrated with quotations. Convergences and divergences between healthcare professionals and the general public’s perspectives are highlighted throughout. Domains and themes are summarized in Table 2.

**Table 2:** Overview of analytic domains and themes developed through reflexive thematic analysis. Domains were deductively informed by the study’s aims and interview structure, while themes within each domain were developed inductively through engagement with the data.

Analytic domain	Themes	Participant quotation
**Domain 1: Conceptualizations of cognitive frailty**	Theme 1.1: Cognitive frailty as a precursor to dementia	*“I would probably think of it as that stage before something more serious develops, like when things are starting to go wrong, but it’s not quite dementia yet.” (Participant 4, general public, man, age 23)* *“It’s probably something that sits before a formal diagnosis, like an early warning stage.” (Participant 14, healthcare professional, woman, age 28)*
	Theme 1.2: Emphasis on cognitive decline over physical frailty	*“I’ve never heard it until this study.” (Participant 1, general public, man, age 23)* *“I guess people focus more on the cognitive side because that’s what stands out clinically.” (Participant 17, healthcare professional, woman, age 29)*
**Domain 2: Perceived manifestations of cognitive frailty **	Theme 2.1: Physical precedes cognitive decline or vice versa	*“If you’re less physically active, your cognitive functions probably decrease as well.” (Participant 5, general public, woman, age 25)* *“You could have physical decline first, which then impacts cognition, but it’s not always a clear sequence.” (Participant 11, healthcare professional, woman, age 53)*
	Theme 2.2: Heterogeneous symptom recognition across groups	*“They both definitely strongly influence each other.” (Participant 9, general public, man, age 30)* *“It’s not just memory, it’s attention, processing speed, and executive functioning as well.” (Participant 13, healthcare professional, woman, age 37)*
**Domain 3: Perceived risk factors for cognitive frailty**	Theme 3.1: Psychological well-being as a mediator of risk	*“Depression would probably be a big risk factor because it affects everything else.” (Participant 4, general public, man, age 23)* *“Depression adds to the overall burden and can accelerate both physical and cognitive decline.” (Participant 16, healthcare professional, woman, age 38)*
	Theme 3.2: Social withdrawal as a gateway to decline	*“If someone stops socialising, that’s when things start to go downhill.” (Participant 6, general public, woman, age 22)* *“Lack of social interaction reduces stimulation and can contribute to decline over time.” (Participant 12, healthcare professional, woman, age 42)*
	Theme 3.3: Lifestyle behaviors as modifiable levers	*“Things like diet and exercise probably make a big difference over time.” (Participant 8, general public, woman, age 26)* *“Lifestyle factors like physical activity and cardiovascular health are key modifiable risks.” (Participant 21, healthcare professional, man, age 33)*

*Note.* Participants first described their understanding of cognitive frailty; the international definition was then provided to support a shared reference point for subsequent questions.

### Domain 1: Conceptualizations of cognitive frailty

**Figure 1:**
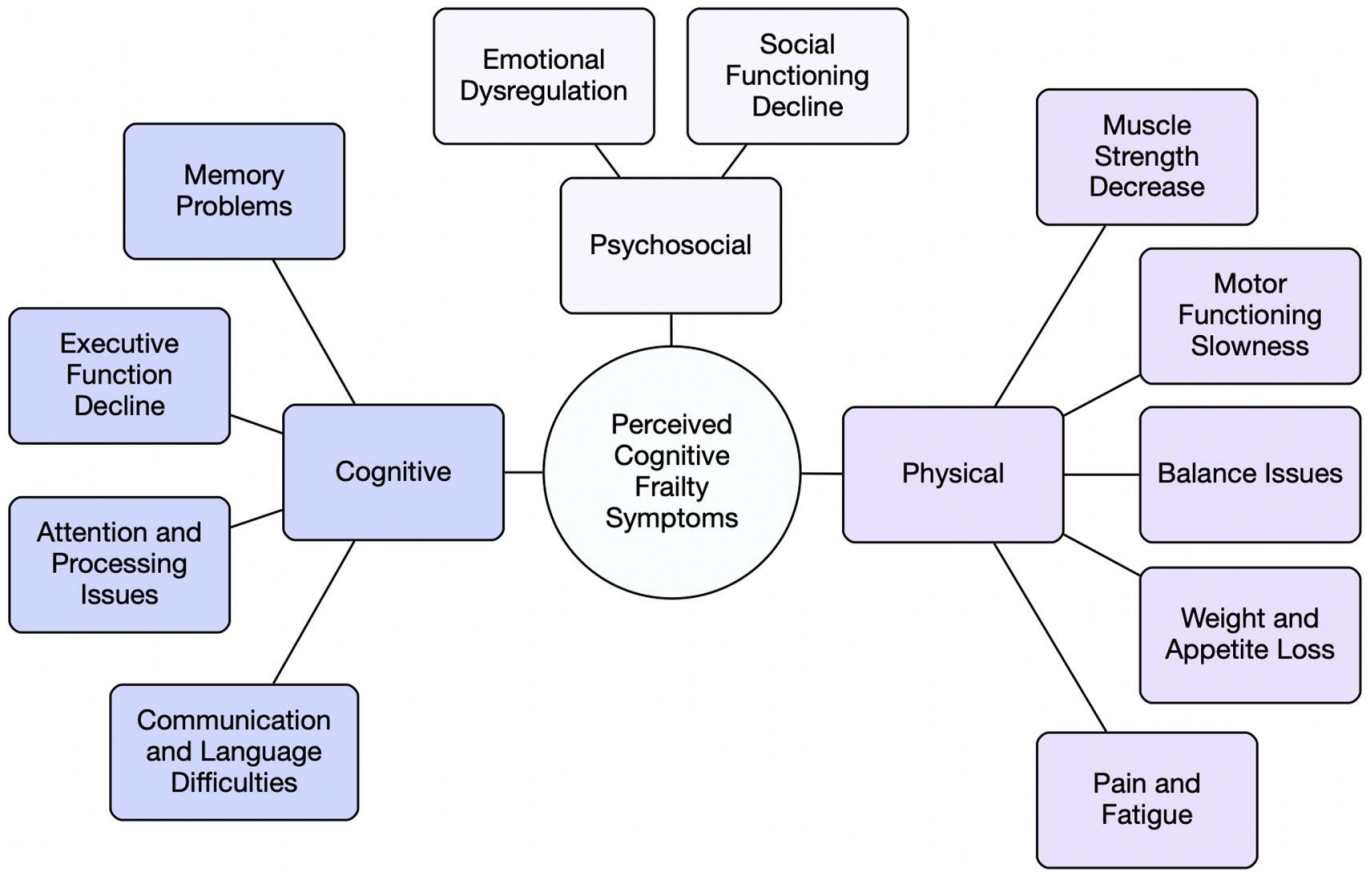
Participant-derived symptom domains for cognitive frailty, based on thematic clustering across interviews.

This domain captures how participants understood the meaning and boundaries of cognitive frailty, before and after being presented with an international definition. Across both groups, cognitive frailty was described as arising from interacting biological, psychological, social, and lifestyle influences. These interrelated domains are visually represented in Figure 1, which maps participants’ conceptualizations of symptoms of cognitive frailty. Two themes were identified: cognitive frailty as an early indicator of dementia, and an emphasis on cognitive decline over physical vulnerability.

#### Theme 1.1: Cognitive frailty as a precursor to dementia

Both healthcare professionals and the general public commonly conceptualized cognitive frailty as an early indicator of decline and positioned it on a continuum leading toward cognitive impairment or dementia, rather than as a distinct syndrome. Several healthcare professionals noted that not all individuals would necessarily progress to dementia, while still describing cognitive frailty as a warning sign:

It’s probably a bit more common than dementia because, in my mind, not all people who are cognitively frail progress towards dementia or a proper cognitive impairment.

(Participant 13, healthcare professional, woman, age 37)

Participants from the general public were generally less familiar with the term but similarly linked it to dementia risk, often framing it as an early decline that might or might not progress, with limited recognition of the dual cognitive and physical nature of the concept.

#### Theme 1.2: Emphasis on cognitive decline over physical frailty

Cognitive decline was often assumed to be the defining feature of cognitive frailty, with limited spontaneous reference to physical frailty, particularly among the general public. Participants commonly foregrounded memory loss and confusion:

I thought it was just the mental aspect of [cognitive frailty] because it has the word 'cognitive’ in it. I assumed this was about cognition specifically.

(Participant 6, general public, woman, age 22)

Even when both domains were acknowledged, the cognitive component was typically prioritized, sometimes with physical frailty treated as secondary or implicit:

A state where someone is experiencing physical frailty or cognitive decline. You know, when one is actually down or having issues with remembering things like memory loss, difficulty focusing, or concentrating.

(Participant 8, general public, woman, age 26)

Overall, the tendency to frame cognition first suggests a bias toward interpreting cognitive frailty through a neurological lens, which may impede recognition of its physical dimension.

### Domain 2: Perceived manifestations of cognitive frailty

This domain reflects how participants described the symptoms and presentation of cognitive frailty, including perceived sequencing of cognitive and physical changes. Two themes were identified: perceived ordering of physical and cognitive decline, and differences in the breadth and specificity of symptom recognition between healthcare professionals and the general public.

#### Theme 2.1: Physical precedes cognitive decline or vice versa

Many participants, particularly healthcare professionals, suggested physical frailty may precede cognitive decline, often attributing this to the visibility of physical changes in everyday functioning and the relative subtlety of early cognitive changes. Some participants cautioned against a linear model and described substantial individual variability. Several participants from the general public similarly emphasized visibility:

Physical one is visible, but cognitive is not visible to the naked eye, so I think physical comes first. It gets noticed and diagnosed more.

(Participant 2, general public, man, age 30)

Others described physical and cognitive changes as concurrent or mutually reinforcing, highlighting the need for integrated recognition and support.

#### Theme 2.2: Heterogeneous symptom recognition across groups

Memory loss was the most consistently cited symptom across both groups. Participants also described concentration and attention problems along with physical manifestations such as weakness, fatigue, and balance difficulties. Healthcare professionals tended to describe a broader and more differentiated cognitive profile, including executive dysfunction and slowed processing:

So, memory lapses, some occasional word-finding difficulties, …slowing down of information processing. Executive functions, attention, concentration, and multitasking would be affected as well.

(Participant 22, healthcare professional, woman, age 55)

By contrast, participants from the general public more often emphasized observable motor and coordination changes:

Those kinds of things, issues with maybe coordination, hand-eye coordination, being a bit slower to move.

(Participant 7, general public, woman, age 23)

Emotional changes and communication difficulties were mentioned less frequently overall but were more commonly raised by healthcare professionals, suggesting that different interpretive frames shaped by clinical versus lay experience were at play.

### Domain 3: Perceived risk factors for cognitive frailty

**Figure 2:**
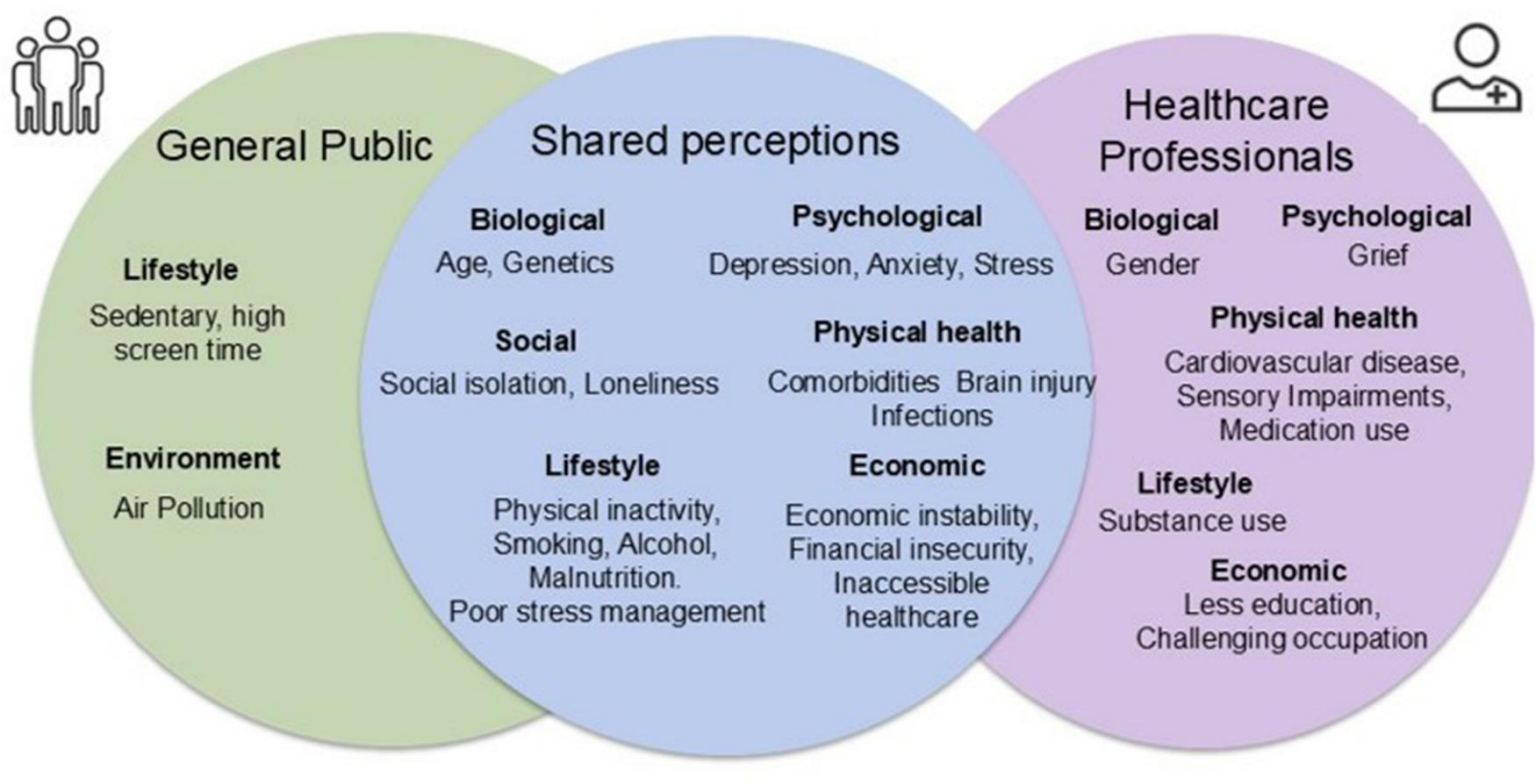
Participant-derived conceptual model illustrating shared and group-specific perceptions of cognitive frailty risk factors among members of the general public and healthcare professionals. Domains reflect factors spontaneously discussed during interviews and should not be interpreted as empirically weighted risk pathways.

This domain captures the risk factors participants linked to cognitive frailty. Both groups described multidimensional influences, including psychological well-being, social engagement, lifestyle behaviors, physical health, and biological aging. Differences between healthcare professionals and the general public primarily reflected differences in emphasis and depth of explanation rather than in wholly distinct sets of risk factors (see Table 3 and Figure 2).

**Table 3:** The perceived risk factors for cognitive frailty checkmarks indicate factors discussed by the group, while cross marks indicate those not discussed.

Risk cluster	Risk factors mentioned	Perceived by	
		**General public**	**Healthcare professionals**
**Lifestyle**	Physical inactivity	✔	✔
	Smoking	✔	✔
	Alcohol consumption	✔	✔
	Substance use	✗	✔
	Malnutrition	✔	✔
	High screen time	✔	✗
	Poor stress management	✔	✔
**Physical health**	Comorbidities	✔	✔
	Cardiovascular diseases	✗	✔
	Physical and brain injuries	✔	✔
	Sensory impairments	✗	✔
	Medication use	✗	✔
	Infections	✔	✔
**Social**	Social isolation	✔	✔
	Loneliness	✔	✔
**Biological**	Genetics	✔	✔
	Age	✔	✔
	Gender	✗	✔
**Psychological**	Depression	✔	✔
	Anxiety	✔	✔
	Grief	✗	✔
	Chronic stress	✔	✔
	Reduced motivation for physical activity	✔	✔
	Diminished engagement in social life	✔	✔
**Economical**	Employment instability	✔	✔
	Less education	✗	✔
	Too challenging an occupation	✔	✔
	Financial insecurity	✔	✔
	Inaccessible healthcare	✔	✔
**Environmental**	Air pollution	✔	✗

*Note.* Factors not mentioned should not be interpreted as perceived absence of risk, but rather as areas not spontaneously raised by participants during interviews.

#### Theme 3.1: Psychological well-being as a mediator of risk

Across both groups, low mood, depression, and anxiety were described as increasing vulnerability to cognitive frailty, often through reduced motivation for self-care, social participation, and healthy behaviors.

There was a shared recognition that poor psychological well-being, particularly depression and anxiety, increased vulnerability to cognitive frailty. Many participants viewed mood as a mediating factor that affects self-care and social activity, and in turn, amplifies broader health risks.

Healthcare professionals often link these mechanisms to biological processes, including brain chemistry, inflammation, and cardiovascular strain. These interpretations tended to draw on clinical knowledge of overlapping risk factors for dementia and physical frailty. One healthcare professional articulated this cascading pathway clearly.

Besides the brain chemistry aspect, there are other factors. Not having the interest to go out and socialise can impact your ability to care for yourself and affect your interest in nutrition and food. So, I would suspect that it increases the risk.

(Participant 12, healthcare professional, woman, age 42)

#### Theme 3.2: Social withdrawal as a gateway to decline

Social disengagement was widely viewed as a key risk factor, linked to reduced cognitive stimulation, poorer mental well-being, and decreased physical activity:

I think people decline a lot more in general when they’re not socially engaged, like being isolated—keeping the mind working is important to maintaining mental health and cognitive health.

(Participant 10, general public, man, age 23)

Healthcare professionals often described social support as enabling protective behaviors and participation in stimulating activities:

…Peer support and having people who understand what you’re going through can really help. Going out and being outside, as well as moving around, obviously helps with various physical aspects. It also encourages communication, so you’re using parts of the brain that you might not be using as much when you’re at home, such as when you’re sitting, watching TV, or listening to the radio. Being in a stimulating environment makes a big difference. People can advise you on different activities, like exercise, community classes, or the arts, whatever helps lift your mood and make you more physically and mentally active. That’s going to be really helpful.

(Participant 18, healthcare professional, woman, age 53)

Social engagement was also described as a cross-cutting domain that facilitates other protective behaviors, such as leaving the house, participating in activities, or maintaining a structured lifestyle.

#### Theme 3.3: Lifestyle behaviors as modifiable levers

Lifestyle behaviors, particularly physical activity and nutrition, were consistently described as modifiable contributors. Participants from the general public tended to emphasize concrete habits, while healthcare professionals more often linked behaviors to physiological mechanisms, including cardiovascular health:

My knowledge is around dementia, but it’s likely a similar set of risk factors. We know that poor nutrition and poor heart health can potentially cause cognitive impairments. I would think they would also directly impact cognitive frailty.

(Participant 11, healthcare professional, woman, age 53)

Some participants also described the cumulative effects of smoking and alcohol misuse:

I can imagine that someone who has spent a lifetime smoking and drinking heavily might not prioritise taking care of themselves. I think these factors all have a direct impact.

(Participant 11, healthcare professional, woman, age 53)

A minority view suggested some behaviors might indirectly support social engagement, highlighting perceived trade-offs between behavioral and social domains.

Together, these themes reflect a shared understanding that cognitive frailty is shaped by modifiable influences. However, the language, framing, and depth of explanation varied markedly across professional and public perspectives.

## Discussion

This study explored how members of the public and healthcare professionals conceptualize cognitive frailty, including its perceived manifestations and risk factors. By comparing these groups, our findings highlight where misunderstandings arise and how they may influence recognition, help-seeking, and intervention. Overall, clearer and more confident language about cognitive frailty may be a necessary precondition for screening, communication, and uptake of risk-reduction strategies.

### Understanding cognitive frailty: perceptions versus literature

Our study found that cognitive frailty is often conceptualized as a precursor to dementia, aligning with its association with increased dementia risk (Shimada et al., 2018, 2018; Zheng et al., 2020). Participants in both groups tended to place cognitive frailty along a dementia continuum, often interpreting it as a prodromal or transitional stage rather than a distinct syndrome in its own right. However, cognitive frailty was rarely recognized as a precursor to disability (Bu et al., 2021; Zhang et al., 2022) and was frequently conflated with MCI, rather than being understood as a condition characterized by the co-occurrence of cognitive vulnerability and physical frailty (Kelaiditi et al., 2013). This reflects a lack of intuitive differentiation, as seen in Domain 1, where participants emphasized cognitive decline and dementia risk but only minimally acknowledged physical frailty. Importantly, participants’ understanding became more differentiated once the international definition was introduced, suggesting that current ambiguity may stem less from conceptual complexity and more from limited exposure to consistent definitions.

The conflation of cognitive frailty with MCI is noteworthy because MCI is more widely recognized and often used as a catch-all term for pre-dementia states. This underscores the limited visibility of cognitive frailty as a public health concept. Greater awareness of its multidimensional nature, especially its physical component, may support earlier recognition and more tailored intervention. Dementia-centric interpretations may narrow attention to cognitive symptoms, underplay physical frailty, and delay interventions targeting mobility, mood, social engagement, and functional resilience.

Participants also differed in their views regarding the temporal sequencing of symptoms. Healthcare professionals more commonly suggested that physical frailty precedes cognitive decline, echoing Ruan et al.'s (2015) proposition that cognitive vulnerability may emerge downstream of physical frailty. In contrast, members of the public were divided, with some describing cognitive and physical decline as simultaneous or mutually reinforcing. Interestingly, although professionals tended to believe that physical symptoms appeared first, physical frailty was mentioned only briefly across interviews, reinforcing a disconnect between knowledge and narrative emphasis. This mismatch matters because if cognitive frailty is discussed primarily as cognitive decline, opportunities for early recognition through physical changes may be missed, and interventions may default to cognitively oriented approaches rather than integrated strategies.

### Symptom recognition: visibility, expertise, and interpretive frames

Memory problems were the most frequently cited symptom across both groups, despite evidence that executive dysfunction, attentional deficits, emotional changes, and social difficulties may be more strongly linked with cognitive frailty (Bunce et al., 2019; Delrieu et al., 2016; Kelaiditi et al., 2013). This reliance on memory as a go-to symptom suggests a conceptual overlap with dementia, potentially limiting broader recognition of cognitive frailty’s multidimensional nature. It may also contribute to delayed identification if individuals interpret early non-memory changes as unrelated to brain health or aging risk.

Healthcare professionals demonstrated a broader, more nuanced recognition of cognitive symptoms, including difficulties with executive function, language, and processing speed, reflecting their clinical experience. In contrast, members of the public relied more heavily on observable and embodied signs, particularly slowed movement, coordination difficulties, and physical decline. These differences show that public participants described symptoms in terms of visibility and lived experience, whereas professionals drew on diagnostic and neurocognitive frameworks. While healthcare professionals were able to identify a broader range of cognitive symptoms, this reflects their diagnostic training and professional exposure. In contrast, public participants, especially younger adults, demonstrated more limited symptom recognition, likely shaped by indirect or limited experience with cognitive frailty. This gap raises concerns about delayed help-seeking in community settings, where early signs may be misattributed or overlooked entirely. A practical implication is that public-facing messaging may need to name and normalize a broader range of early cognitive changes beyond memory and explicitly link them to physical vulnerability and function.

Within the physical domain, participants identified muscle weakness, fatigue, weight loss, and balance problems, consistent with the physical frailty literature (Fried et al., 2001). However, spontaneous integration of physical and cognitive features was rare, reinforcing the finding that the dual nature of cognitive frailty remains poorly internalized. These discrepancies in symptom recognition highlight the need for targeted education strategies that bridge clinical knowledge and public understanding. Such strategies should support both recognition and communication, particularly given evidence that some clinicians may avoid using the term “frailty” because of its negative connotations (Griffin et al., 2024).

### Perceived risk factors: multidimensional but unevenly integrated

Participants identified a wide range of perceived risk factors for cognitive frailty, spanning lifestyle, physical health, psychological well-being, social engagement, biological processes, economic circumstances, and environmental factors. This aligns closely with the risk clusters summarized in Table 3 and with recent multidimensional models of cognitive frailty risk (Fowler-Davis et al., 2024a, 2024b; Hodgson et al., 2023; Holland et al., 2024; Mitchell, 2009). However, participants differed in how strongly they connected these factors into causal or reinforcing pathways, with professionals more likely to articulate their interdependence.

Healthcare professionals articulated more interconnected explanatory pathways, particularly linking cardiovascular health, mental well-being, and lifestyle behaviors. For example, they drew parallels between dementia risk and cognitive frailty, citing smoking, alcohol use, and nutrition as common drivers. Members of the public, by contrast, tended to focus on fewer domains, often emphasizing mood, activity levels, or environmental context. This divergence suggests that while public understanding captures key risk elements, it may lack an integrative framework connecting these factors. This matters because fragmented risk beliefs can reduce perceived controllability and diminish motivation for sustained risk-reduction behaviors.

Low mood emerged as a central mediator across groups, with participants describing how depression or anxiety reduced motivation for self-care, social engagement, and healthy behaviors. This mediating role of psychological well-being was one of the most consistent findings across analytic domains and aligns with emerging evidence on the cascading effects of mental health on cognitive and physical decline (Holland et al., 2024). Social withdrawal was also widely recognized as a gateway to further decline, with participants describing reduced cognitive stimulation, physical inactivity, and emotional deterioration. Healthcare professionals were more likely to distinguish between social engagement and social support, highlighting the protective role of meaningful relationships. These findings reinforce the role of social connectedness as a cross-cutting protective factor that links psychological well-being, lifestyle behaviors, and cognitive reserve. Together, these accounts support intervention approaches that treat mood and social connectedness as core components of prevention and management rather than as secondary consequences.

The disparity between professional and public recognition of cognitive frailty also has implications for targeted education. The young age profile of many public participants in this study offered valuable insight into emerging generational narratives but may also reflect a lack of lived experience with cognitive decline. It is plausible that older individuals in the general population, particularly those with caregiving experience, may demonstrate a more nuanced understanding, underscoring the need for future research to compare perspectives across the life course. Future work could also purposively recruit people with direct experience of frailty, MCI, or caregiving to examine how proximity to aging-related health needs reshapes mental models of cognitive frailty and perceived preventability.

### Tackling cognitive frailty: a comprehensive, collaborative approach

Our findings suggest that communication, shared understanding, and conceptual clarity are necessary preconditions for screening, workforce development, and prevention. Misconceptions persisted in both lay and professional narratives, particularly around the integration of physical and cognitive domains. Standardized tools may benefit from integrating cognitive, physical, psychological, and social components, but such efforts are likely to be most effective when preceded by clearer communication among professionals and the public. Brief, accessible explanations could be embedded in primary care and community services to support consistent language and reduce conceptual drift.

Public health messaging that aligns with international definitions and foregrounds the dual cognitive–physical nature of cognitive frailty may help reduce confusion, counter fatalistic narratives, and support earlier recognition by distinguishing cognitive frailty from MCI, which has itself been variably framed and subtyped within clinical discourse (Bradfield, 2023; Kelaiditi et al., 2013; Ruan et al., 2015). Framing cognitive frailty as modifiable rather than inevitable may help counter fatalism and stigma, encouraging proactive engagement with prevention strategies (Guzmán et al., 2021; Lim et al., 2024; Sánchez-García et al., 2017; Schoenborn et al., 2018). Notably, in our interviews, providing a definition prompted many participants to reframe cognitive frailty as more preventable and actionable, suggesting that message framing could directly influence perceived agency.

Cognitive frailty offers a distinct window for integrated intervention across cognitive and physical domains. Unlike MCI-focused approaches, which often prioritize cognitive stimulation or dementia-delay strategies, cognitive frailty requires multimodal responses addressing both physical frailty and cognitive vulnerability. Multidomain trials such as the Finnish Geriatric Intervention Study to Prevent Cognitive Impairment and Disability (FINGER) and the Multidomain Alzheimer Preventive Trial (MAPT) demonstrate the potential benefits of combined lifestyle, cognitive, nutritional, and vascular risk approaches in at-risk older adults (Andrieu et al., 2017; Ngandu et al., 2015), while the Active Geriatric Evaluation for Longevity and Lifestyle for Elderly Study (AGELESS) targets cognitive frailty specifically (Ponvel et al., 2021). However, many trials recruited individuals with or at risk of MCI rather than cognitive frailty as a defined syndrome. Existing evidence, therefore, supports the promise of multidomain approaches but cannot be directly extrapolated to cognitive frailty. In contrast, the Systolic Blood Pressure Intervention Trial, Memory and Cognition in Decreased Hypertension (SPRINT MIND) trial, which focused solely on vascular risk reduction, showed more modest cognitive benefits and did not directly address physical frailty (Williamson et al., 2019). Taken together, these findings suggest that although evidence on existing interventions cannot be directly extrapolated to cognitive frailty, it supports the potential value of multidomain approaches for populations with overlapping profiles of cognitive and physical vulnerability. This underscores the need for interventions that explicitly address the dual cognitive–physical nature of cognitive frailty, rather than relying on strategies developed for cognitive impairment or dementia in isolation.

Translating these multidomain interventions into real-world settings requires more than clinical evidence; it calls for proactive engagement, workforce development, and supportive environments that enable sustained behavior change. Initiatives that support social engagement, mental well-being, and healthy living should be prioritized as preventive strategies, especially for those at risk. Our findings indicate that equipping healthcare professionals with clearer language and conceptual frameworks for cognitive frailty may support more confident recognition and communication, thereby facilitating appropriate referral, prevention, and intervention pathways. Given the reluctance to use the term frailty in practice (Griffin et al., 2024), training should also include language options that preserve accuracy while reducing stigma and fatalism.

For healthcare professionals, training should focus on recognizing cognitive frailty, communicating it sensitively, and addressing patients’ perceptions of the condition (González-Mariscal et al., 2025; Griffin et al., 2024). Programs targeting low mood, social isolation, and healthy behaviors may be important for prevention and management (Aguilar-Navarro et al., 2025; Sugimoto et al., 2022). At the policy level, addressing social determinants such as healthcare accessibility, social isolation, economic hardship, and environmental factors remains essential (Fowler-Davis et al., 2024b; Hodgson et al., 2023; Zheng et al., 2022). Overall, improving conceptual clarity may be a practical lever for earlier identification and greater engagement with integrated prevention strategies.

## Strengths and limitations

To our knowledge, this is the first qualitative study to explore perceptions of cognitive frailty among both healthcare professionals and members of the public. Reflexive thematic analysis enabled an in-depth examination of spontaneous and prompted interpretations of a concept unfamiliar to many. Comparing two groups helped identify knowledge gaps and differing interpretive frameworks across levels of health literacy and professional exposure.

Nonetheless, several limitations should be acknowledged. The use of purposive, snowball, and convenience sampling may have introduced selection bias, and participants may not fully represent the wider diversity of healthcare professionals or the general population. In particular, the public group skewed toward younger adults, which may limit the extent to which the findings reflect the perspectives of older adults at greatest risk of cognitive frailty. However, this inclusion allowed exploration of how early-life beliefs and social narratives shaped the understanding of cognitive health risks across the life course.

The sample size (*N* = 22) is also a consideration. While small, it was adequate given the study’s narrow aim, the specificity of participant groups, and the richness of the interview data. Using the concept of information power (Malterud et al., 2016), we argue that the adequacy of the sample lies in the depth of information obtained rather than quantity. Within-group thematic saturation was achieved, and the dual-group design enabled meaningful comparisons despite the relatively small number of participants. We acknowledge that the small sample may affect the transferability (rather than generalizability) of findings to other contexts. Given the reflexive thematic analysis approach, we treat transferability as supported by rich description and analytic transparency rather than by representativeness.

While the gender distribution was relatively balanced in the public group, the healthcare professional sample included a greater proportion of women. That said, interpretations of cognitive frailty did not appear to differ by gender. Finally, although participants were provided with a working definition of cognitive frailty to anchor discussions, this could have influenced their views. To minimize this potential bias, open-ended questions were used initially to capture participants’ preexisting interpretations before introducing the definition. This sequencing ensured that participant-led understandings were not overly shaped by the researchers’ framing. At the same time, the observed shift after definition provision is informative in itself, as it mirrors the likely impact of brief explanations in routine practice.

## Conclusion

Our findings show that cognitive frailty is frequently interpreted through a dementia-centered lens, particularly among younger members of the general public, with limited recognition of its physical component and broader multidimensional risk profile. Healthcare professionals demonstrated greater awareness of symptom complexity and interrelated risk factors, whereas public participants relied more heavily on visible and embodied cues, reflecting differences in exposure and experience. Across both groups, psychological well-being, social engagement, and lifestyle behaviors emerged as central and modifiable influences, although their interdependence was more clearly articulated by professionals. These findings suggest that cognitive frailty is not yet established as a shared or consistently understood construct across public and professional contexts. This lack of shared understanding may limit its translation from research into routine clinical practice, public health messaging, and prevention initiatives. To advance earlier recognition and prevention, there is a need to strengthen conceptual clarity and shared understanding of cognitive frailty across public and clinical contexts. Aligning language with international definitions and foregrounding the construct’s dual cognitive–physical nature may help shift cognitive frailty from an abstract research concept to an actionable focus for prevention and care, with improved communication, therefore viewed not as an endpoint, but as a critical enabling step for earlier recognition, more appropriate risk reduction, and integrated approaches that support independence and quality of life in later life.

## Supplementary Material

gnag112_Supplementary_Data

## Data Availability

Due to the sensitive nature of the qualitative interview data and restrictions imposed by the Research Ethics Committee, the full dataset cannot be made publicly available in order to protect participant confidentiality. Anonymized excerpts relevant to the study findings are included within the manuscript. The analytic procedures are described in sufficient detail to support transparency and reproducibility. This study was not preregistered.

## Patient and public involvement

Patients and/or the public were involved in the design, conduct, reporting, or dissemination plans of this research. Further details are provided in the Methods section.

## References

[gnag112-B1] Aguilar-Navarro S. G. , Mimenza-Alvarado A. J., Yeverino-Castro S. G., Caicedo-Correa S. M., Cano-Gutiérrez C. (2025). Cognitive frailty and aging: Clinical characteristics, pathophysiological mechanisms, and potential prevention strategies. Archives of Medical Research, 56, 103106. 10.1016/j.arcmed.2024.103106 39522432

[gnag112-B3] Andrieu S. , Guyonnet S., Coley N., Cantet C., Bonnefoy M., Bordes S., Bories L., Cufi M.-N., Dantoine T., Dartigues J.-F., Desclaux F., Gabelle A., Gasnier Y., Pesce A., Sudres K., Touchon J., Robert P., Rouaud O., Legrand P., Vellas B., MAPT Study Group. (2017). Effect of long-term omega 3 polyunsaturated fatty acid supplementation with or without multidomain intervention on cognitive function in elderly adults with memory complaints (MAPT): A randomised, placebo-controlled trial. The Lancet. Neurology, 16, 377–389. 10.1016/S1474-4422(17)30040-6 28359749

[gnag112-B6] Ballenger J. F. (2006a). Progress in the history of Alzheimer’s disease: The importance of context. Journal of Alzheimer’s Disease: JAD, 9, 5–13. 10.3233/JAD-2006-9S302 17004361

[gnag112-B7] Ballenger J. F. (2006b). Self, senility, and Alzheimer’s disease in modern America: A history. Johns Hopkins University Press. https://www.press.jhu.edu/books/title/3725/self-senility-and-alzheimers-disease-modern-america?srsltid=AfmBOopO8mHz6rA16Pd-NI3yr177k2Vaw8-H9zLyjLv09fuLod4w5Wre

[gnag112-B8] Ballenger J. F. (2017). Framing confusion: Dementia, society, and history. AMA Journal of Ethics, 19, 713–719. 10.1001/journalofethics.2017.19.7.mhst1-1707 28813244

[gnag112-B9] Belder C. R. S. , Schott J. M., Fox N. C. (2023). Preparing for disease-modifying therapies in Alzheimer’s disease. The Lancet. Neurology, 22, 782–783. 10.1016/S1474-4422(23)00274-0 37463598

[gnag112-B10] Bradfield N. I. (2023). Mild cognitive impairment: Diagnosis and subtypes. Clinical EEG and Neuroscience, 54, 4–11. 10.1177/15500594211042708 34549629

[gnag112-B11] Braun V. , Clarke V. (2019). Reflecting on reflexive thematic analysis. Qualitative Research in Sport, Exercise and Health, 11, 589–597. 10.1080/2159676X.2019.1628806

[gnag112-B12] Breton A. , Casey D., Arnaoutoglou N. A. (2019). Cognitive tests for the detection of mild cognitive impairment (MCI), the prodromal stage of dementia: Meta-analysis of diagnostic accuracy studies. International Journal of Geriatric Psychiatry, 34, 233–242. 10.1002/gps.5016 30370616

[gnag112-B13] Bu Z. , Huang A., Xue M., Li Q., Bai Y., Xu G. (2021). Cognitive frailty as a predictor of adverse outcomes among older adults: A systematic review and meta-analysis. Brain and Behavior, 11, e01926. 10.1002/brb3.1926 33159430 PMC7821586

[gnag112-B14] Bunce D. , Batterham P. J., Mackinnon A. J. (2019). Long-term associations between physical frailty and performance in specific cognitive domains. The Journals of Gerontology. Series B, Psychological Sciences and Social Sciences, 74, 919–926. 10.1093/geronb/gbx177 29401240

[gnag112-B15] Delrieu J. , Andrieu S., Pahor M., Cantet C., Cesari M., Ousset P. J., Voisin T., Fougère B., Gillette-Guyonnet S., Carrie I., Vellas B. (2016). Neuropsychological profile of “cognitive frailty” subjects in the MAPT study. The Journal of Prevention of Alzheimer’s Disease, 3, 151–159. 10.14283/jpad.2016.94 PMC4991881 27547746

[gnag112-B16] Emmady P. D. , Tadi P. (2022). Major neurocognitive disorder (dementia). StatPearls Publishing. https://www.ncbi.nlm.nih.gov/books/NBK557444/32491376

[gnag112-B17] Fowler Davis S. , Benkowitz C., Holland C., Gow A., & Clarke C. (2024). A Scoping Review on the Opportunities for Social Engagement and Cognitive Frailty in Older Adults. *Public Health Reviews*, 45, 1606494 DOI: 10.3389/phrs.2024.1606494 PMC: 38389543 38389543 PMC10882720

[gnag112-B18] Fowler-Davis S. , Benkowitz C., Nield L., Dayson C. (2024b). Green spaces and the impact on cognitive frailty: A scoping review. Frontiers in Public Health, 11, 1278542. 10.3389/fpubh.2023.1278542 PMC10810992 38283295

[gnag112-B19] Fried L. P. , Tangen C. M., Walston J., Newman A. B., Hirsch C., Gottdiener J., Seeman T., Tracy R., Kop W. J., Burke G., McBurnie M. A., Cardiovascular Health Study Collaborative Research Group. (2001). Frailty in older adults: Evidence for a phenotype. The Journals of Gerontology. Series A, Biological Sciences and Medical Sciences, 56, M146–M156. 10.1093/gerona/56.3.M146 11253156

[gnag112-B20] González-Mariscal A. , Corral-Pérez J., Vázquez-Sánchez M. Á., Ávila-Cabeza-de-Vaca L., Costilla M., Casals C. (2025). Benefits of an educational intervention on functional capacity in community-dwelling older adults with frailty phenotype: A randomised controlled trial. International Journal of Nursing Studies, 162, 104955. 10.1016/j.ijnurstu.2024.104955 39579605

[gnag112-B21] Griffin N. , O’Sullivan L., Usher R. (2024). Frailty: Perceptions of occupational therapists in Ireland. Irish Journal of Occupational Therapy, 52, 36–43. 10.1108/IJOT-08-2023-0018

[gnag112-B22] Guzmán A. , Gillanders D., Stevenson A., Ross K. (2021). Psychosocial adjustment to Mild Cognitive Impairment: The role of illness perceptions, cognitive fusion and cognitive impairment. Dementia (London, England), 20, 464–484. 10.1177/1471301219893862 31948271

[gnag112-B23] Hodgson J. R. , Benkowitz C., Castellani B. C., Ellison A., Yassaie R., Twohig H., Bhudia R., Jutila O.-E. I., Fowler-Davis S. (2023). A scoping review of the effects of ambient air quality on cognitive frailty. Environments, 11, 4. 10.3390/environments11010004

[gnag112-B24] Holland C. , Dravecz N., Owens L., Benedetto A., Dias I., Gow A., Broughton S. (2024). Understanding exogenous factors and biological mechanisms for cognitive frailty: A multidisciplinary scoping review. Ageing Research Reviews, 101, 102461. 10.1016/j.arr.2024.102461 39278273

[gnag112-B26] Hanipah J. M. , Mat F., Kaur D., Subramaniam P., Shahar S. (2024). Limited health literacy increases the likelihood of cognitive frailty among older adults. BMC Geriatrics, 24(1), 840. 10.1186/s12877-024-05419-x PMC11475880 39407098

[gnag112-B27] Kelaiditi E. , Cesari M., Canevelli M., Abellan van Kan G., Ousset P.-J., Gillette-Guyonnet S., Ritz P., Duveau F., Soto M. E., Provencher V., Nourhashemi F., Salva A., Robert P., Andrieu S., Rolland Y., Touchon J., Fitten J. L., Vellas B. (2013). Cognitive frailty: Rationale and definition from an (I.A.N.A./I.A.G.G.) International Consensus Group. The Journal of Nutrition, Health & Aging, 17, 726–734. 10.1007/s12603-013-0367-2 PMC12878905 24154642

[gnag112-B28] Kmet L. M. , Cook L. S., Lee R. C. (2004). *Standard quality assessment criteria for evaluating primary research papers from a variety of fields*. 10.7939/R37M04F16

[gnag112-B29] Lim S. H. , Østbye T., Seah V. Q. H., Aloweni F. (2024). Exploring perceptions of frailty, resilience, and self-efficacy in older adults and caregivers in acute care context. Research in Nursing & Health, 47, 39–48. 10.1002/nur.22353 37982359

[gnag112-B30] Malterud K. , Siersma V. D., Guassora A. D. (2016). Sample size in qualitative interview studies: Guided by information power. Qualitative Health Research, 26, 1753–1760. 10.1177/1049732315617444 26613970

[gnag112-B32] Mitchell A. J. (2009). A meta-analysis of the accuracy of the mini-mental state examination in the detection of dementia and mild cognitive impairment. Journal of Psychiatric Research, 43, 411–431. 10.1016/j.jpsychires.2008.04.014 18579155

[gnag112-B33] Mitchell A. J. , Shiri-Feshki M. (2009). Rate of progression of mild cognitive impairment to dementia: Meta-analysis of 41 robust inception cohort studies. Acta Psychiatrica Scandinavica, 119, 252–265. 10.1111/j.1600-0447.2008.01326.x 19236314

[gnag112-B34] Murukesu R. R. , Singh D. K. A., Shahar S., Subramaniam P. (2020). A multi-domain intervention protocol for the potential reversal of cognitive frailty: “WE-RISE” randomized controlled trial. Frontiers in Public Health, 8, 471. 10.3389/fpubh.2020.00471 33014971 PMC7495818

[gnag112-B35] Nader M. M. , Cosarderelioglu C., Miao E., Whitson H., Xue Q.-L., Grodstein F., Oh E., Ferrucci L., Bennett D. A., Walston J. D., George C., Abadir P. M. (2023). Navigating and diagnosing cognitive frailty in research and clinical domains. Nature Aging, 3, 1325–1333. 10.1038/s43587-023-00504-z 37845509 PMC10936574

[gnag112-B37] Ngandu T. , Lehtisalo J., Solomon A., Levälahti E., Ahtiluoto S., Antikainen R., Bäckman L., Hänninen T., Jula A., Laatikainen T., Lindström J., Mangialasche F., Paajanen T., Pajala S., Peltonen M., Rauramaa R., Stigsdotter-Neely A., Strandberg T., Tuomilehto J., Kivipelto M. (2015). A 2-year multidomain intervention of diet, exercise, cognitive training, and vascular risk monitoring versus control to prevent cognitive decline in at-risk elderly people (FINGER): A randomised controlled trial. Lancet (London, England), 385, 2255–2263. 10.1016/S0140-6736(15)60461-5 25771249

[gnag112-B39] Panza F. , Lozupone M., Solfrizzi V., Sardone R., Dibello V., Di Lena L., D’Urso F., Stallone R., Petruzzi M., Giannelli G., Quaranta N., Bellomo A., Greco A., Daniele A., Seripa D., Logroscino G. (2018). Different cognitive frailty models and health- and cognitive-related outcomes in older age: From epidemiology to prevention. Journal of Alzheimer’s Disease: JAD, 62, 993–1012. 10.3233/JAD-170963 29562543 PMC5870024

[gnag112-B40] Parish A. , Kim J., Lewallen K. M., Miller S., Myers J., Panepinto R., Maxwell C. A. (2019). Knowledge and perceptions about aging and frailty: An integrative review of literature. Geriatric Nursing (New York, N.Y.), 40, 13–24. 10.1016/j.gerinurse.2018.05.007 29909928

[gnag112-B43] Ponvel P. , Shahar S., Singh D. K. A., Ludin A. F. M., Rajikan R., Rajab N. F., Ai-Vyrn C., Din N. C., Ibrahim N., Subramaniam P., Haron H., Ismail A., Sharif R., Ramasamy K., Majeed A. B. A., Ali N. M., Mohamad M., Noah S. A. M., Ibrahim A. M., Kivipelto M. (2021). Multidomain intervention for reversal of cognitive frailty, towards a personalized approach (AGELESS trial): Study design. Journal of Alzheimer’s Disease: JAD, 82, 673–687. 10.3233/JAD-201607 PMC8385532 34092633

[gnag112-B44] Profyri E. , Leung P., Huntley J., Orgeta V. (2022). Effectiveness of treatments for people living with severe dementia: A systematic review and meta-analysis of randomised controlled clinical trials. Ageing Research Reviews, 82, 101758. 10.1016/j.arr.2022.101758 36243355 PMC10580243

[gnag112-B45] Rivan N. F. M. , Shahar S., Singh D. K. A., Che Din N., Mahadzir H., You Y. X., Kamaruddin M. Z. A. (2024). Development of cognitive frailty screening tool among community-dwelling older adults. Heliyon, 10, e34223. 10.1016/j.heliyon.2024.e34223 39104490 PMC11298820

[gnag112-B47] Rosenberg C. E. , Golden J., Peitzman S. J. (1992). Framing disease. Hospital Practice (Office ed.), 27, 179–221. 10.1080/21548331.1992.11705460

[gnag112-B48] Ruan Q. , Yu Z., Chen M., Bao Z., Li J., He W. (2015). Cognitive frailty, a novel target for the prevention of elderly dependency. Ageing Research Reviews, 20, 1–10. 10.1016/j.arr.2014.12.004 25555677

[gnag112-B49] Sánchez-García S. , Gallegos-Carrillo K., Espinel-Bermúdez M. C., Doubova S. V., Sánchez-Arenas R., García-Peña C., Salvà A., Briseño-Fabian S. C. (2017). Comparison of quality of life among community-dwelling older adults with the frailty phenotype. Quality of Life Research: An International Journal of Quality of Life Aspects of Treatment, Care and Rehabilitation, 26, 2693–2703. 10.1007/s11136-017-1630-5 28667436

[gnag112-B50] Schoenborn N. L. , Van Pilsum Rasmussen S. E., Xue Q.-L., Walston J. D., McAdams-Demarco M. A., Segev D. L., Boyd C. M. (2018). Older adults’ perceptions and informational needs regarding frailty. BMC Geriatrics, 18, 46. 10.1186/s12877-018-0741-3 29433426 PMC5809948

[gnag112-B51] Shafiq S. , Haith-Cooper M., Hawkins R., Parveen S. (2023). What are lay UK public perceptions of frailty: A scoping review. Age and Ageing, 52(4), afad045. 10.1093/ageing/afad045 PMC10066724 37002930

[gnag112-B52] Shimada H. , Doi T., Lee S., Makizako H., Chen L.-K., Arai H. (2018). Cognitive frailty predicts incident dementia among community-dwelling older people. Journal of Clinical Medicine, 7, 250. 10.3390/jcm7090250 30200236 PMC6162851

[gnag112-B53] Shimada H. , Makizako H., Tsutsumimoto K., Doi T., Lee S., Suzuki T. (2018). Cognitive frailty and incidence of dementia in older persons. The Journal of Prevention of Alzheimer’s Disease, 5, 42–48. 10.14283/jpad.2017.29 PMC12280713 29405232

[gnag112-B54] Sugimoto T. , Arai H., Sakurai T. (2022). An update on cognitive frailty: Its definition, impact, associated factors, underlying mechanisms, and interventions. Geriatrics & Gerontology International, 22, 99–109. 10.1111/ggi.14322 34882939

[gnag112-B55] Sugimoto T. , Sakurai T., Ono R., Kimura A., Saji N., Niida S., Toba K., Chen L. K., Arai H. (2018). Epidemiological and clinical significance of cognitive frailty: A mini review. Ageing Research Reviews, 44, 1–7. 10.1016/j.arr.2018.03.002 29544875

[gnag112-B56] Tseng S.-H. , Liu L.-K., Peng L.-N., Wang P.-N., Loh C.-H., Chen L.-K. (2019). Development and validation of a tool to screen for cognitive frailty among community-dwelling elders. The Journal of Nutrition, Health & Aging, 23, 904–909. 10.1007/s12603-019-1235-5 PMC12280652 31641743

[gnag112-B57] United Nations, Department of Economic and Social Affairs, Population Division. (2024). *World population prospects 2024: Summary of results* (UN DESA/POP/2024/TR/NO. 9). https://population.un.org/wpp/assets/Files/WPP2024_Summary-of-Results.pdf

[gnag112-B60] Williamson J. D. , Pajewski N. M., Auchus A. P., Bryan R. N., Chelune G., Cheung A. K., Cleveland M. L., Coker L. H., Crowe M. G., Cushman W. C., Cutler J. A., Davatzikos C., Desiderio L., Erus G., Fine L. J., Gaussoin S. A., Harris D., Hsieh M.-K., Wright C. B. (2019). Effect of intensive vs standard blood pressure control on probable dementia: A randomized clinical trial. JAMA, 321, 553–561. 10.1001/jama.2018.21442 30688979 PMC6439590

[gnag112-B62] World Health Organization. (2024). *Ageing and health*. https://www.who.int/news-room/fact-sheets/detail/ageing-and-health

[gnag112-B63] Zhang X. M. , Wu X. J., Cao J., Jiao J., Chen W. (2022). Association between cognitive frailty and adverse outcomes among older adults: A meta-analysis. The Journal of Nutrition, Health & Aging, 26, 817–825. 10.1007/s12603-022-1833-5 PMC12280716 36156673

[gnag112-B64] Zheng L. , Li G., Gao D., Wang S., Meng X., Wang C., Yuan H., Chen L. (2020). Cognitive frailty as a predictor of dementia among older adults: A systematic review and meta-analysis. Archives of Gerontology and Geriatrics, 87, 103997. 10.1016/j.archger.2019.103997 31846833

[gnag112-B65] Zheng L. , Wang C., Qiu Y., Li X., Zhang X., Zhang M., Ma T., Li G., Chen L. (2022). Effectiveness of interventions in older adults with cognitive frailty: A systematic review and meta-analysis of randomised controlled trials. Age and Ageing, 51(12), afac286. 10.1093/ageing/afac286 36571775

