# Supplementary material for: Unravelling cognitive frailty: perceptions, misconceptions, and the path to prevention
Source: Gerontologist. 2026 May 26;66(7):gnag112. doi: 10.1093/geront/gnag112 (PMC13329062; doi:10.1093/geront/gnag112)
Supplement: gnag112_Supplementary_Data [file gnag112_supplementary_data.pdf]

Unravelling Cognitive Frailty: Perceptions, Misconceptions, and the Path to Prevention

Gerda Prakopimaite, Magdalena Pfaff, Eddy J. Davelaar, Lisa Quadt, Malgorzata Raczek, Naji Tabet, Alan J. Gow, Carol Holland, Dorina Cadar

Supplementary material 1. Topic guide for semi-structured interviews

Opening	Age, gender, education, occupation, and role.<br><br>For healthcare professionals, whether they work with people at risk of frailty or not, the length of time in their current role.
Understanding of the term 'cognitive frailty'	What is your understanding of the term 'frailty'?<br><br>Have you ever heard of the term 'cognitive frailty'?<br>- If yes, in what context have you heard the term 'cognitive frailty'?<br><br>What do you think cognitive frailty is?<br><br>**Participants were then provided with the definition of cognitive frailty:** the co-occurrence of mild cognitive impairment and physical frailty without the diagnosis of dementia or Alzheimer's.<br><br>How common do you think cognitive frailty is?<br><br>At what age do you think cognitive frailty occurs?<br><br>Do you think cognitive frailty is independent of physical frailty?
Cognitive frailty symptoms	Do you think cognitive decline or physical decline comes first?<br><br>How do you think cognitive frailty manifests?

Risks associated with cognitive frailty	What do you think are the main risk factors associated with cognitive frailty?<br><br>To what extent do you think … could contribute to the likelihood of developing cognitive frailty?<br>- Low mood, such as anxiety or depression?<br>- Low social engagement and social support?<br>- Lifestyle factors such as low physical activity, smoking, and drinking?
Personal experience with cognitive frailty	Do you think you have ever experienced mild cognitive impairment?<br><br>Do you think you have ever experienced physical frailty?<br><br>Do you think you have ever experienced cognitive frailty?<br><br>Is there anything else you would like to share?
